# LINC01116 promotes proliferation and migration of endometrial stromal cells by targeting FOXP1 via sponging miR‐9‐5p in endometriosis

**DOI:** 10.1111/jcmm.16039

**Published:** 2020-12-28

**Authors:** Liangyi Cui, Silei Chen, Dandan Wang, Qing Yang

**Affiliations:** ^1^ Department of Obstetrics & Gynecology Shengjing Hospital of China Medical University Shenyang China

**Keywords:** ceRNA, endometriosis, FOXP1, LINC01116, miR‐9‐5p

## Abstract

Endometriosis is a common multi‐factorial gynaecological disease. Recent studies have revealed that long non‐coding RNAs (lncRNAs) are involved in the pathogenesis of endometriosis. In the present study, the expression profiles of lncRNAs in 6 pairs of endometriosis ectopic endometrium (ecEM) and eutopic endometrium (euEM) tissues were analysed by RNA sequencing. From the profiles, LINC01116 was found to be up‐regulated in ecEM tissues compared to euEM tissues and was verified by quantitative real‐time PCR (qRT‐PCR). Then, functional experiments demonstrated that LINC01116 promoted the proliferation and migration of ectopic primary endometrial stromal cells (ESCs), while miR‐9‐5p exerted the opposite effects. Dual‐luciferase reporter assays verified that LINC01116 directly sponged miR‐9‐5p and relieved the suppression of its target, Forkhead box protein P1 (FOXP1). Rescue experiments further demonstrated that LINC01116 could promote proliferation and migration of ESCs by targeting FOXP1 via sponging miR‐9‐5p. Overall, our study illuminates that LINC01116 promotes the progression of endometriosis through the miR‐9‐5p/FOXP1 axis. This finding provides a novel therapeutic target for patients with endometriosis.

## 1 INTRODUCTION

Endometriosis is a common gynaecologic disorder, which affects 6 to 10% of women of reproductive age. It refers to the abnormal growth of functional endometrial tissue (glands and stroma) outside the uterine cavity.^1^, ^2^, ^3^ The most common symptoms of endometriosis include dysmenorrhoea, chronic pelvic pain and infertility.^4^, ^5^ Although endometriosis is a benign gynaecological disease, it does have biological behaviours that are similar to malignant tumours, such as tissue invasion, local spread, distant metastasis and recurrence.^1^ Unfortunately, there are still no early diagnostic tools or radical treatments for this disease owing to the diversity of its symptoms and complexity of its pathogenesis.^6^, ^7^ Therefore, the most urgent task is to deeply investigate the pathophysiological basis of this disease.^8^, ^9^

Long non‐coding RNAs (lncRNAs) refer to RNAs that contain more than 200 nucleotides but have low protein encoding potential.^10^, ^11^ Mounting evidence has shown that lncRNAs are involved in various diseases in the human body, including cancer, cardiovascular diseases and autoimmune diseases.^12^, ^13^, ^14^ At present, many studies have revealed that lncRNAs are involved in the occurrence and development of endometriosis.^15^, ^16^, ^17^, ^18^ For instance, lncRNA AFAP1‐AS1 promoted the epithelial‐mesenchymal transition (EMT) in endometriosis and was correlated with the transcription factor, ZEB1.^15^ Furthermore, down‐regulation of lncRNA H19 inhibited endometriosis invasion and migration through sponging miR‐216a‐5p.^16^ This evidence indicates that lncRNAs may play an important role in the cellular pathophysiological process of endometriosis.

Long intergenic non‐coding RNA 01 116 (LINC01116), also known as TALNEC2, is located in the 2q31.1 region of human chromosome. LINC01116 has been reported to act as both an oncogene and a tumour suppressor gene depending on the tumour type, such as in breast cancer,^19^ osteosarcoma,^20^, ^21^ ovarian cancer,^22^ glioma,^23^, ^24^ head and neck squamous carcinoma,^25^ gastric cancer^26^ and lung cancer.^27^ However, the specific role of LINC01116 in endometriosis has yet to be investigated.

In the present study, we explored lncRNA expression profiles in 6 paired endometriosis eutopic endometrium (euEM) and ectopic endometrium (ecEM) tissues using high‐throughput RNA sequencing. We found LINC01116 was significantly up‐regulated in ecEM tissues. We aimed to investigate the function and mechanism of LINC01116 in endometriosis in vitro. This is the first time that the LINC01116/miR‐9‐5p/Forkhead box protein P1 (FOXP1) axis has been explored in endometriosis.

## 2 MATERIALS AND METHODS

### 2.1 Clinical specimens

This study was approved by the Institutional Ethics Review Board of ShengJing Hospital of the China Medical University and all patients signed informed consent forms. EuEM and ecEM tissues were collected from patients (22‐46 years old) with rASRM (the Revised American Society for Reproductive Medicine classification system, 1997) stage III/IV ovarian endometriosis that was diagnosed laparoscopically and by histopathology at ShengJing Hospital of the China Medical University from March 1, 2018 to January 31, 2019. All enrolled patients had regular menstruation periods (21‐35 days) and had not received any sexual hormone therapy for at least 6 months before their operation.

### 2.2 Library preparation and lncRNA sequencing

Each sample required 3 μg RNA for preparation. After removing the ribosomal RNA, the rRNA‐depleted RNA by NEBNext^®^ Ultra™ Directional RNA Library Prep Kit for Illumina^®^ (NEB) was used to generate sequencing libraries according to the manufacturer's instructions. The products were purified and assessed by Agilent Bioanalyzer 2100 system. After cluster generation, the libraries were sequenced on an Illumina Hiseq 4000 platform and 150 bp paired‐end reads were generated. After quality control, mapping to reference genome, transcriptome assembly and coding potential analysis were performed, transcripts predicted without coding potential were our candidate set of lncRNAs. The RNA‐Seq and data analysis were performed by Novogene Co. LTD.

### 2.3 Differential expression analysis

Cuffdiff (v2.1.1) was used to calculate the FPKMs (number of Fragments Per Kilobase of transcript sequence per Millions base pairs sequenced) of transcripts in each sample.^28^ Gene FPKMs were calculated by summing up the FPKMs of the transcripts in each gene group. Cuffdiff provides statistical routines for determining differential expression in digital transcripts or gene expression data using a model based on the negative binomial distribution. The differentially expressed lncRNAs were defined as |log fold change (FC)| >2 and *P*‐value <.01.

### 2.4 Primary endometrial stromal cell isolation and culture

Isolation of primary endometrial stromal cells (ESCs) was performed according to previously reported methods.^29^ Briefly, ectopic endometrial tissues were washed with phosphate‐buffered saline (PBS) and digested with DMEM/F‐12(BI, Israel) medium containing type I collagenase (2 mg/mL) (Sigma‐Aldrich) in an agitated water bath at 37°C for 1.5 hours. The cell suspensions were then filtered by 400‐μm cell‐strainers (Falcon). After that the filtrates were centrifugated at 230 ×*g*  for 5 minutes, washed with PBS, and resuspended in DMEM/F‐12 medium supplemented with 10% foetal bovine serum (ExCell Biology), 100 μg/mL streptomycin, and 100 U/mL penicillin. ESCs were then plated in 25‐cm^2^ flasks and cultured at 37°C in 5% CO_2_. The ESCs were used during the 2‐6 passage. The purity of the ESCs was examined by Immunofluorescence of vimentin (a stromal cell marker) (1:200, Proteintech, 60330‐1‐Ig).

### 2.5 RNA extraction and qRT‐PCR

Total RNA of the tissues was extracted by using Trizol reagent (Takara Bio) according to the manufacturer's protocol. The RNA concentration was measured by a N50 Touch (Implen) Spectrophotometer. The lncRNA and mRNA were converted into cDNA using the PrimeScript™ RT reagent Kit with gDNA Eraser (Takara Bio). The miRNA was converted into cDNA using the miRNA 1st Strand cDNA Synthesis Kit (Stem‐Loop, Vazyme). The qRT‐PCR for the mRNA and lncRNA was performed using TB Green™ Premix Ex Taq™ (Takara Bio) with GAPDH as an internal control. The qRT‐PCR for miRNA was performed using the miRNA Universal SYBR^®^ qPCR Master Mix (Vazyme) with small nuclear RNA U6 as an internal control. The relative gene expression level was calculated using the 2^−ΔΔCt^ method. The primer sequences are listed in Table 1.

**TABLE 1:** List of primer sequences for qRT‐PCR

Primer name	Sequence (5ʹ‐3ʹ)
LINC01116 Forward	GGAGTGTGGTGGCACCATCATG
LINC01116 Reverse	AGCTACTCGGAAGGCTGAGGTG
LINC00473 Forward	TCCACGGAGGTCTTAAGGCAGAG
LINC00473 Reverse	CGTCAGAAGGAGGAGCAGGTAGG
LINC01018 Forward	TGCGGTGGAAGTCTTAGAGTAGCC
LINC01018 Reverse	CTGCTGACACTGCTGTGCTGAC
ADAMTS9‐AS1 Forward	CTCAGACCACAACTCTCCACCTTG
ADAMTS9‐AS1 Reverse	CAGATGCTGCCTGGCTGATGG
GATA2‐AS1 Forward	AGGCTGGGATGATGGTGGAGAC
GATA2‐AS1 Reverse	CCGTGAAGATGGTGCTGGGAATG
FOXP1 Forward	TCCAGAAAAGCAGCTAACACTA
FOXP1 Reverse	TTCTACTCGCACAAAACACTTG
GAPDH Forward	CAGGAGGCATTGCTGATGAT
GAPDH Reverse	GAAGGCTGGGGCTCATTT
hsa‐miR‐744‐5p Forward	GTGCGGGGCTAGGGCTA
hsa‐miR‐744‐5p Reverse	AGTGCAGGGTCCGAGGTATT
hsa‐miR‐744‐5p RT Primer	GTCGTATCCAGTGCAGGGTCCGAGGTATT CGCACTGGATACGACTCATAC
hsa‐miR‐650 Forward	CGAGGAGGCAGCGCTCT
hsa‐miR‐650 Reverse	AGTGCAGGGTCCGAGGTATT
hsa‐miR‐650 RT Primer	GTCGTATCCAGTGCAGGGTCCGAGGTATT CGCACTGGATACGACGTCCTG
hsa‐miR‐9‐5p Forward	GCGCGTCTTTGGTTATCTAGCT
hsa‐miR‐9‐5p Reverse	AGTGCAGGGTCCGAGGTATT
hsa‐miR‐9‐5p RT Primer	GTCGTATCCAGTGCAGGGTCCGAGGTATTC GCACTGGATACGACTCATAC
hsa‐miR‐31‐5p Forward	ACACTCCAGCTGGGAGGCAAGATGCTGGC
hsa‐miR‐31‐5p Reverse	TGGTGTCGTGGAGTCG
hsa‐miR‐31‐5p RT Primer	GTCGTATCCAGTGCAGGGTCCGAGGTAT TCGCACTGGATACGACTGGGGT
hsa‐miR‐3612 Forward	GCGAGGAGGCATCTTGAGA
hsa‐miR‐3612 Reverse	AGTGCAGGGTCCGAGGTATT
hsa‐miR‐3612 RT Primer	GTCGTATCCAGTGCAGGGTCCGAGGT ATTCGCACTGGATACGACTCCATT
hsa‐U6 Forward	CGCTTCGGCAGCACATATAC
hsa‐U6 Reverse	TTCACGAATTTGCGTGTCATC
hsa‐U6 RT Primer	AAAATATGGAACGC

### 2.6 Construction of lentivirus

In order to suppress the expression of LINC01116 in ESCs, the following shRNA and scrambled control (NC) shRNA were inserted into the GV112 vector : sh‐LINC01116‐1 5’‐GAGCAGTGTATTAGAAGACAA‐3’, sh‐LINC01116‐2 5’‐TAGAGACCGAGTCTCAACTAT‐3’, sh‐LINC01116‐3 5’‐TCGCTTTGCTGAAGACGAGCA‐3’, sh‐NC 5’‐TTCTCCGAACGTGTCACGT‐3’. Lentivirus packaging was performed by Genecham.

### 2.7 Vector and oligonucleotide transfection

The vector containing the full length of LINC01116 and the empty vector were designed by Syngentech. The miR‐9‐5p mimics, miR‐9‐5p inhibitor and their negative control were purchased from GenePharma, Shanghai, China. ESCs reached 60%‐70% confluence in 6‐well plates before transfection and Lipofectamine 3000 (Invitrogen) was used to transfect vectors and oligonucleotides according to the manufacturer's protocol.

### 2.8 Cell counting kit‐8 proliferation assay

A total of 2000 ESCs were seeded in each well of a 96‐well plate. Ten micro‐litres of CCK‐8 reagent (Bimake) were added at specific timepoints (0, 24, 48, 72 hours), into the culture medium. After incubating for 2 hours at 37°C in 5% CO_2_, the optical density (OD) was measured at 450 nm using a microplate reader (BioTek Instruments). These experiments were done in triplicate.

### 2.9 Wound healing assay

ESCs of each treatment group were cultured in six‐well plates, and cell monolayer was subsequently scratched with a 200‐µL pipette tip. Cell culture was performed using DMEM/F12 supplemented with 1% FBS to reduce the effect of cell proliferation. Representative images were captured by a microscope (Nikon) at 0 and 24 hours after injury. For each one, the experiments were repeated for three times.

### 2.10 Trans‐well migration assay

The trans‐well chamber (8‐μM pore size trans‐well filter) was utilized to detect cell migration. Cells were diluted to a density of 1 × 10^5^/mL in 200 μL serum‐free medium in the upper chambers, while the lower chambers were filled with 700 μL complete culture medium as the chemoattractant. After 24 hours, the upper chambers were fixed with 4% paraformaldehyde and then stained with 0.1% crystal violet. The stained migrated cells on the membrane were photographed using a microscope (Nikon). These experiments were performed in triplicate.

### 2.11 Dual‐luciferase reporter assay

The 3′‐UTR sequences of LINC01116 and FOXP1 containing the wild‐type or mutant miR‐9‐5p binding sites were synthesized and inserted into pmirGLO luciferase reporter vectors (Promega) and co‐transfected into ESCs with miR‐9‐5p mimics or mimicsNC using Lipofectamine 3000. After 48 hours of cell transfection, the cells were lysed and assayed for luciferase activity using the Dual‐Glo Luciferase Assay System (Promega) according to the manufacturer's instructions. Firefly luciferase activity was normalized to Renilla luciferase activity and was expressed as a percentage of the control. These experiments were performed in triplicate.

### 2.12 Western blotting

Total proteins of tissues and cells were extracted using RIPA reagent (Beyotime) supplemented with phenylmethanesulphonyl fluoride (PMSF) (Beyotime). The protein concentration was measured using a BCA protein assay kit (Beyotime). Electrophoresis was performed using a 10% PAGE Gel Fast Preparation Kit (EpiZyme). A total of 20 μg protein was added to each well, and a polyvinylidene difluoride (PVDF) membrane (Immobilon) was used for protein transferring. After electrophoresis, the PVDF membrane was blocked in 5% non‐fat milk for 2 hours and then incubated with primary antibody for 12‐16 hours at 4°C. After which, the membrane was washed with Tris‐buffered saline‐Tween (TBST) and incubated with the corresponding secondary antibodies for 2 hours at room temperature, then visualized using the Quantity One imaging software (Bio‐Rad). The blots were visualized using enhanced chemiluminescence reagent (ECL, Beyotime) and the related data were analysed using Image Lab Software. The primary antibodies involved in this analysis were as follows: GAPDH (1:5000, Proteintech, 60004‐1‐Ig), and FOXP1 (1:2000, Proteintech, 22051‐1‐AP).

### 2.13 Statistical analysis

All data were presented as the mean ± standard error (SE) from at least three independent replicates. Statistical analysis and statistical mapping were performed using GraphPad Prism 8 software. Student's *t* test was used to compare whether there is a difference between two sets of normally distributed data. If normality was not obtained, the nonparametric Mann‐Whitney U test was used. A *P* ≤ .05 was considered statistically significant.

## 3 RESULTS

### 3.1 Expression profiles of lncRNAs in euEM and ecEM tissues

**FIGURE 1:**
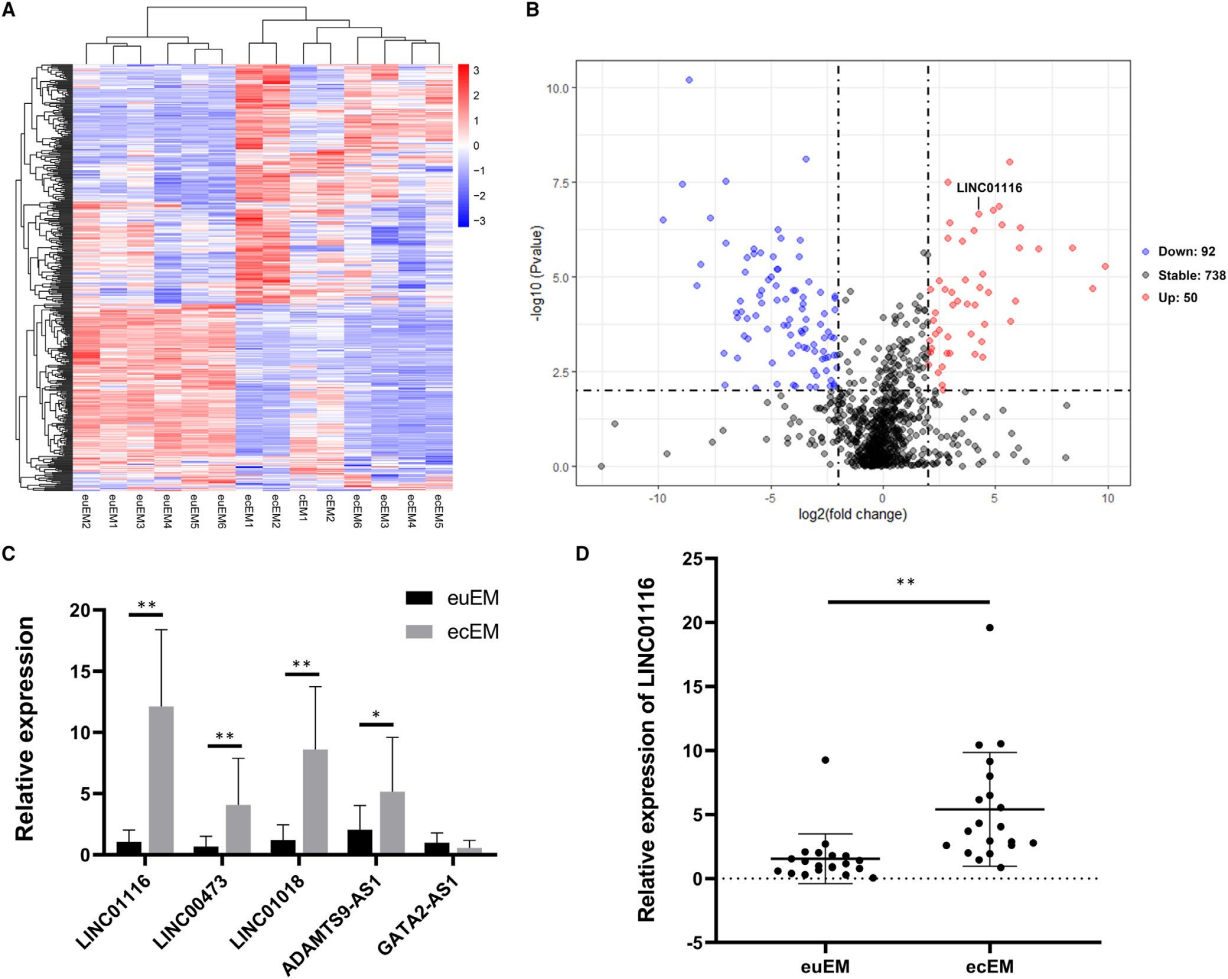
Expression profiles of lncRNAs in endometriosis tissues. A, The significantly dysregulated lncRNAs in ecEM tissues relative to euEM tissues were shown in a cluster heat map. The red and blue strips represent high and low expression, respectively. B, A volcano plot demonstrates differentially expressed lncRNAs between ecEM and euEM tissues. The up‐regulated lncRNAs were in red points while the down‐regulated lncRNAs were in blue points. C, Expression level of 5 lncRNAs in tissues by qRT‐PCR (n = 8). D, Relative expression levels of LINC01116 in euEM and ecEM tissues by qRT‐PCR (n = 20). Data are shown as mean ± SD, *P < .05, **P < .01

To investigate the role of lncRNAs in the development of endometriosis, RNA sequencing was performed on 6 paired euEM and ecEM tissues and a lncRNA profiling database was constructed.^30^ A cluster heat map was used to show the significantly dysregulated lncRNAs in the euEM tissues compared to the paired ecEM tissues (Figure 1A). A total of 142 lncRNAs were defined as DEGs (logFC > 2, *P* < .01), including 50 up‐regulated lncRNAs and 92 down‐regulated lncRNAs (Figure 1B). Top 20 up‐regulated and down‐regulated lncRNAs in ecEM tissues compared with paired euEM tissues were listed in Table 2. Among these DEGs, LINC01116 was found to be significantly up‐regulated (logFC = 3.6685, *P* < .0001). We selected 4 (LINC01116, LINC00473, LINC01018, ADAMTS9‐AS1) up‐regulated and 1 (GATA2‐AS1) down‐regulated lncRNA for qRT‐PCR verification. The results were consistent with our RNA‐seq data (Figure 1C). Furthermore, we detected the expression of LINC01116 in 20 paired euEM and ecEM tissues by RT‐qPCR. We found that the expression of LINC01116 was significantly up‐regulated in ecEM compared with euEM (Figure 1D).

**TABLE 2:** Top 20 up‐regulated and down‐regulated lncRNAs in ectopic endometrium tissues compared with paired eutopic endometrium tissues

Up‐regulated lncRNAs			Down‐regulated lncRNAs		
Gene name	logFC	*P*‐value	Gene name	logFC	*P*‐value
MIR202HG	9.8603	5.24E‐06	RP11‐319E12.2	−9.7912	3.09E‐07
CTD‐2332E11.2	8.4000	1.73E‐06	AC008060.7	−8.9623	3.48E‐08
LEMD1‐AS1	6.9154	1.85E‐06	RP11‐624M8.1	−8.6385	6.21E‐11
ADAMTS9‐AS1	6.0949	4.95E‐07	AC009110.1	−8.1284	4.58E‐06
LINC01018	6.0546	1.75E‐06	RP11‐6E9.4	−7.6890	2.82E‐07
GATA6‐AS1	5.6070	9.40E‐09	RP11‐143E21.3	−7.0304	1.28E‐06
RP11‐834C11.4	5.2947	4.27E‐07	PRICKLE2‐AS1	−7.0004	3.04E‐08
RP11‐615I2.2	5.1569	1.34E‐07	LINC00645	−6.1704	7.62E‐06
RP11‐535A5.1	4.8949	1.70E‐07	RP11‐624M8.1	−6.0646	3.05E‐06
RP11‐366L20.2	4.4080	8.21E‐06	U47924.27	−5.7706	1.79E‐06
AC012531.25	4.2541	2.24E‐07	LINC01480	−5.7509	2.45E‐06
LINC00473	4.0592	5.48E‐05	HNF1A‐AS1	−5.1102	1.21E‐05
RP11‐366L20.2	4.0354	6.10E‐07	RP11‐519G16.5	−4.9065	2.84E‐06
LINC01116	3.6685	1.18E‐05	RP11‐231E6.1	−4.7223	6.19E‐06
RP6‐24A23.3	3.5335	1.15E‐06	LINC01541	−4.6964	6.13E‐06
LINC01197	2.9664	3.73E‐07	RP11‐611O2.5	−4.6897	5.75E‐07
RP11‐400K9.4	2.8858	3.24E‐08	CTD‐2396E7.11	−4.5404	9.46E‐07
RP1‐78O14.1	2.8787	9.46E‐07	GATA2‐AS1	−3.7441	2.89E‐06
PGM5P2	1.9711	2.58E‐06	RP11‐357H14.16	−3.7198	1.08E‐06
DUBR	1.7985	2.31E‐06	BMPR1B‐AS1	−3.4501	7.95E‐09

### 3.2 LINC01116 promotes ESCs proliferation and migration in vitro

**FIGURE 2:**
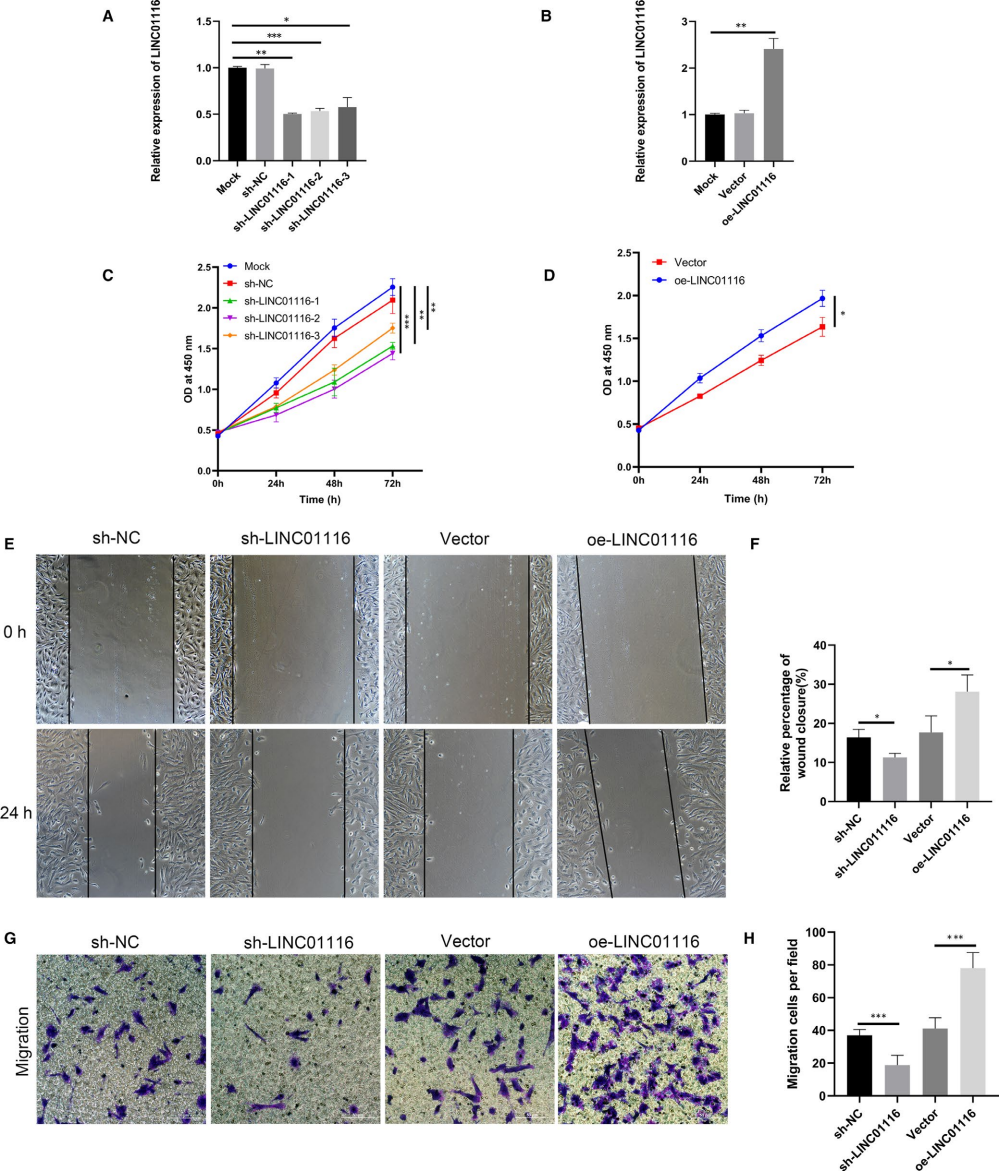
LINC01116 promotes endometrial stromal cell proliferation and migration in vitro. A, The knockdown efficiency of sh‐LINC01116 lentivirus assessed by qRT‐PCR in endometrial stromal cells (ESCs). B, The overexpression of LINC01116 in ESCs confirmed by qRT‐PCR. (C and D) CCK‐8 assays performed to identify the effect of LINC01116 on the proliferative ability of ESCs. (E to H) Wound healing and trans‐well assays performed to identify the effect of LINC01116 on the migrative ability of ESCs. Data are shown as mean ± SD, *P < .05, **P < .01, ***P < .001

To investigate the potential biological function of LINC01116 in ESCs, three lentiviruses (sh‐LINC01116‐1, sh‐LINC01116‐2, sh‐LINC01116‐3) were used to knockdown the expression of LINC01116 (Figure 2A). An overexpression vector (oe‐LINC01116) of LINC01116 was also constructed and transfected into ESCs (Figure 2B). Finally, we choose sh‐LINC01116‐1 for further experiments due to its high efficiency.

To explore the effect of LINC01116 on cell viability, we performed CCK‐8 assays on ESCs after transfecting them with sh‐LINC01116 or oe‐LINC01116. CCK‐8 assays revealed that the knockdown of LINC01116 significantly inhibited the proliferation ability of ESCs, whereas the overexpression of LINC01116 exerted the opposite effect (Figure 2C,D). Wound healing and trans‐well assays showed that the migration capabilities of ESCs were remarkably suppressed by the knockdown of LINC01116 but significantly enhanced by the overexpression of LINC01116 (Figure 2E‐H). These experiments indicated that LINC01116 plays an important role in promoting cell proliferation and migration in endometriosis.

### 3.3 LINC01116 sponges miR‐9‐5p in endometriosis

**FIGURE 3:**
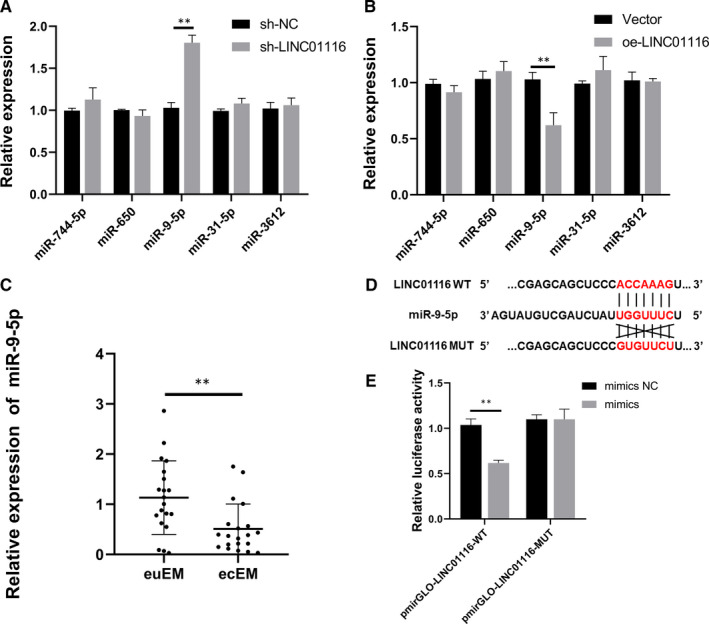
LINC01116 serves as a sponge of miR‐9‐5p. (A and B) Relative expression of 5 miRNAs in ESCs after transfection with sh‐LINC01116 or oe‐LINC01116. C, Relative expression levels of miR‐9‐5p in euEM and ecEM tissues by qRT‐PCR (n = 20). D, The molecular binding site of miR‐9‐5p and WT or MUT LINC01116. (E) Dual‐luciferase reporter assays confirmed the combination between LINC01116 and miR‐9‐5p. Data are shown as mean ± SD, *P < .05, **P < .01

According to the competing endogenous RNA (ceRNA) theory, lncRNAs can sponge miRNAs and regulate their expression in the cytoplasm.^31^, ^32^ Database Starbase (http://starbase.sysu.edu.cn/) was used to predict potential miRNAs that bind with LINC01116. From this database, 5 candidate miRNAs (miR‐744‐5p, miR‐650, miR‐9‐5p, miR‐31‐5p, miR‐3612) were chosen. We examined the expression level of candidate miRNAs in ESCs transfected with sh‐LINC01116 or oe‐LINC01116, only miR‐9‐5p was enhanced in the sh‐LINC01116 group and decreased in the oe‐LINC01116 group (Figure 3A,B). Furthermore, the expression level of miR‐9‐5p was significantly up‐regulated in ecEM relative to euEM tissues by qRT‐PCR (Figure 3C). Finally, the dual‐luciferase reporter assay further demonstrated the direct binding between LINC01116 and miR‐9‐5p (Figure 3D). The luciferase reporter vector GP‐miRGLO containing LINC01116‐WT or LINC01116‐MUT and miR‐9‐5p mimics or mimics NC were co‐transfected into ESCs to determine the interaction between LINC01116 and miR‐9‐5p. The luciferase activity of pmirGLO‐LINC01116‐WT was reduced in mimics group compared to the mimics NC group while there were no such differences observed in pmirGLO‐LINC01116‐MUT (Figure 3E). These data suggest that LINC01116 epigenetically sponges miR‐9‐5p in endometriosis.

### 3.4 MiR‐9‐5p suppresses ESCs proliferation and migration in vitro

**FIGURE 4:**
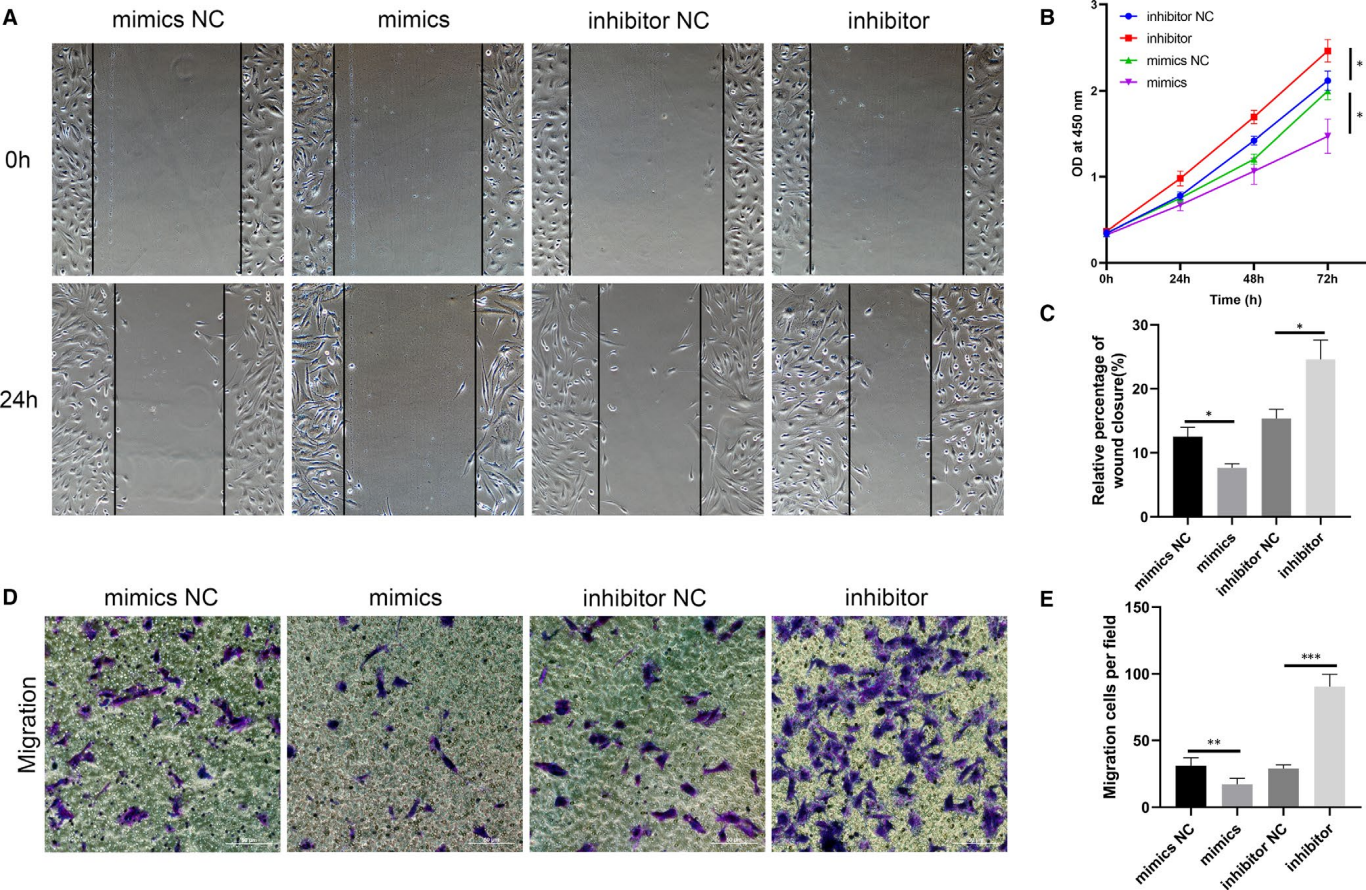
MiR‐9‐5p inhibits ESCs proliferation and migration in vitro. (A, C, D and E) Cell migratory capability tested by wound healing and trans‐well assays in ESCs transfected with miR‐9‐5p mimics, miR‐9‐5p inhibitor, and their NCs. (B) CCK‐8 assays performed to assess the proliferation ability of ESCs transfected with miR‐9‐5p mimics, miR‐9‐5p inhibitor and their NCs. Data are shown as mean ± SD, *P < .05, **P < .01, ***P < .001

Though miR‐9‐5p has been investigated in many other diseases,^33^, ^34^, ^35^ little is known about the role of miR‐9‐5p in endometriosis. We investigated the potential biological function and mechanism of miR‐9‐5p in endometriosis by transfecting miR‐9‐5p mimics, miR‐9‐5p inhibitor and their NC into ESCs. Growth curves of CCK8 assays indicated that miR‐9‐5p mimics weakened the proliferation ability of ESCs, while the miR‐9‐5p inhibitor exerted the opposite effect (Figure 4B). Wound healing and trans‐well assays demonstrated that the migration abilities of ESCs were significantly impaired by miR‐9‐5p mimics but remarkably promoted by the miR‐9‐5p inhibitor (Figure 4A,C‐E).

### 3.5 MiR‐9‐5p directly targets FOXP1 in endometriosis

**FIGURE 5:**
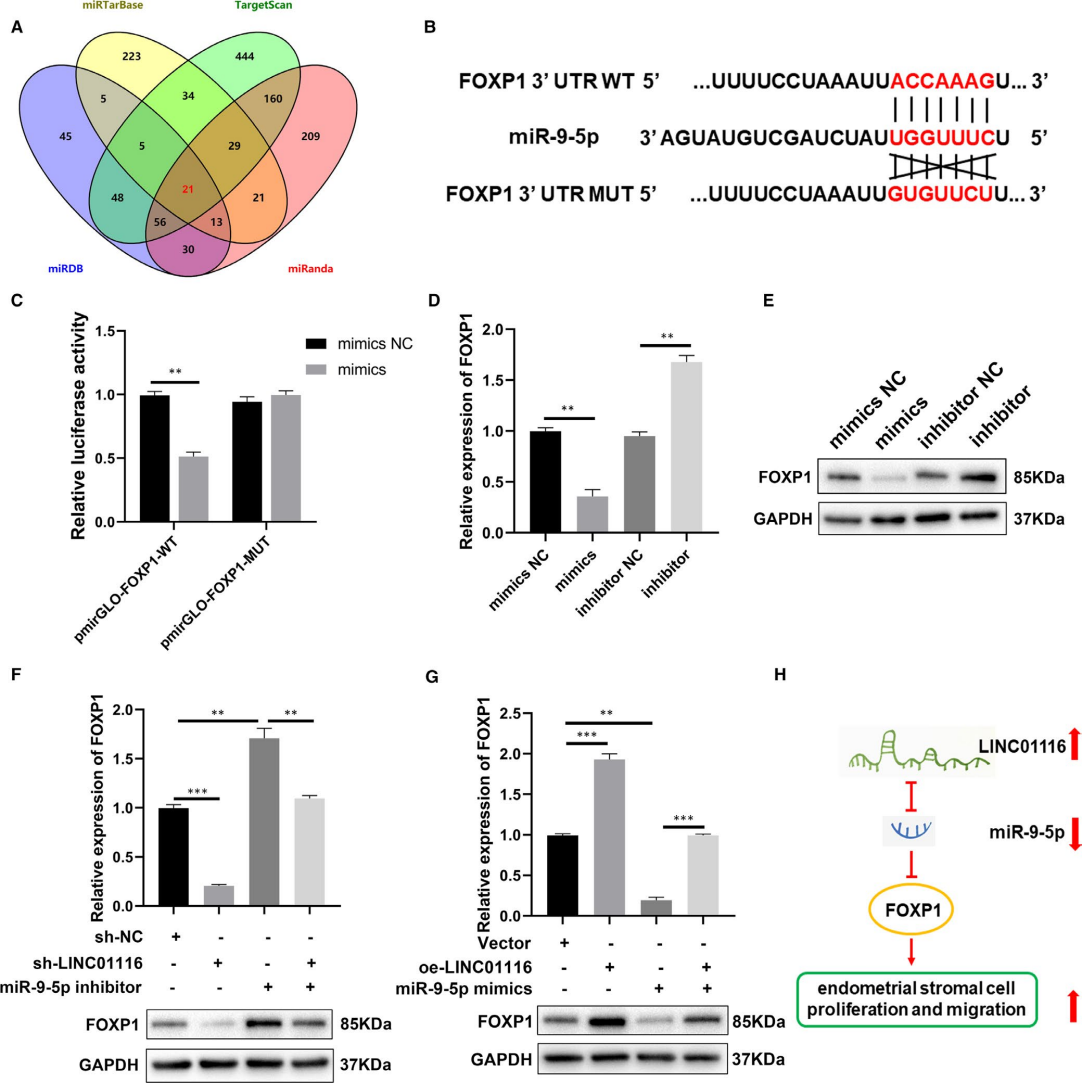
LINC01116 serves as a miRNA sponge of miR‐9‐5p that regulates FOXP1 expression. A, The overlapping of the target genes of miR‐9‐5p predicted by 4 database, miRTarBase, miRDB, miRanda and TargetScan. B, The molecular binding site of miR‐9‐5p and WT or MUT FOXP13’UTR. (C) Dual‐luciferase reporter assays confirmed the combination between miR‐9‐5p and FOXP1. (D and E) Relative mRNA level of FOXP1 evaluated by qRT‐PCR (left) and protein level of FOXP1 evaluated by Western blot (right) in ESCs transfected with the miR‐9‐5p mimics or inhibitor (F and G) Relative mRNA and protein levels of FOXP1 evaluated in ESCs transfected with indicated sh‐NC, sh‐LINC01116, inhibitor, vector, oe‐LINC01116, or mimics by qRT‐PCR (up) and Western blot (down). (H) Schematic diagram of LINC01116/miR‐9‐5p/FOXP1 axis in endometriosis. Data are shown as mean ± SD, *P < .05, **P < .01, ***P < .001

MiRNAs epigenetically regulate gene expression by binding with the 3′UTR of target mRNAs. Here, 4 databases (miRTarBase, miRanda, miRDB and TargetScan) were adopted to predict the potential target genes of miR‐9‐5p. There were 21 common candidate target genes (ONECUT2, LIN28B, SHC1, KLF5, FOXP1, etc) (Figure 5A). Among them, FOXP1 has been reported to improve cell proliferation and enhance fibrosis in endometriosis,^36^, ^37^ thus FOXP1 was selected as miR‐9‐5p's target gene for further study. Subsequently, qRT‐PCR and Western blot showed that miR‐9‐5p mimics reduced the FOXP1 mRNA level as well as the FOXP1 protein levels in ESCs while the miR‐9‐5p inhibitor exerted the opposite effects (Figure 5D,E). Finally, the dual‐luciferase reporter assay was performed to demonstrate the binding site between miR‐9‐5p and FOXP1. GP‐miRGLO vector containing FOXP1‐WT or FOXP1‐MUT and miR‐9‐5p mimics or mimics NC were co‐transfected into ESCs (Figure 5B). The results revealed that the luciferase activity was decreased in the FOXP1‐WT groups compared to in the FOXP1‐MUT groups after co‐transfection of miR‐9‐5p (Figure 5C). Collectively, these results demonstrate that FOXP1 is a target mRNA of miR‐9‐5p in endometriosis.

### 3.6 LINC01116 serves as a miR‐9‐5p sponge that regulates FOXP1 expression

We performed a series of experiments to confirm the interactions between LINC01116, miR‐9‐5p and FOXP1 in endometriosis. The qRT‐PCR and Western blot results revealed that knockdown of LINC01116 significantly decreased the mRNA and protein levels of FOXP1 in ESCs, while the miR‐9‐5p inhibitor increased them. Co‐transfection of sh‐LINC01116 and the miR‐9‐5p inhibitor counteracted these effects in ESCs (Figure 5F). Similarly, overexpression of LINC01116 increased the mRNA and protein levels of FOXP1 whereas miR‐9‐5p mimics had the opposite effect. Co‐transfection of oe‐LINC01116 and miR‐9‐5p mimics also counteracted these effects in ESCs (Figure 5G). Then we considered that LINC01116 promoted the progression of endometriosis via the LINC01116/miR‐9‐5p/FOXP1 axis (Figure 5H).

### 3.7 LINC01116 promotes ESCs proliferation and migration through LINC01116/miR‐9‐5p/FOXP1 axis

**FIGURE 6:**
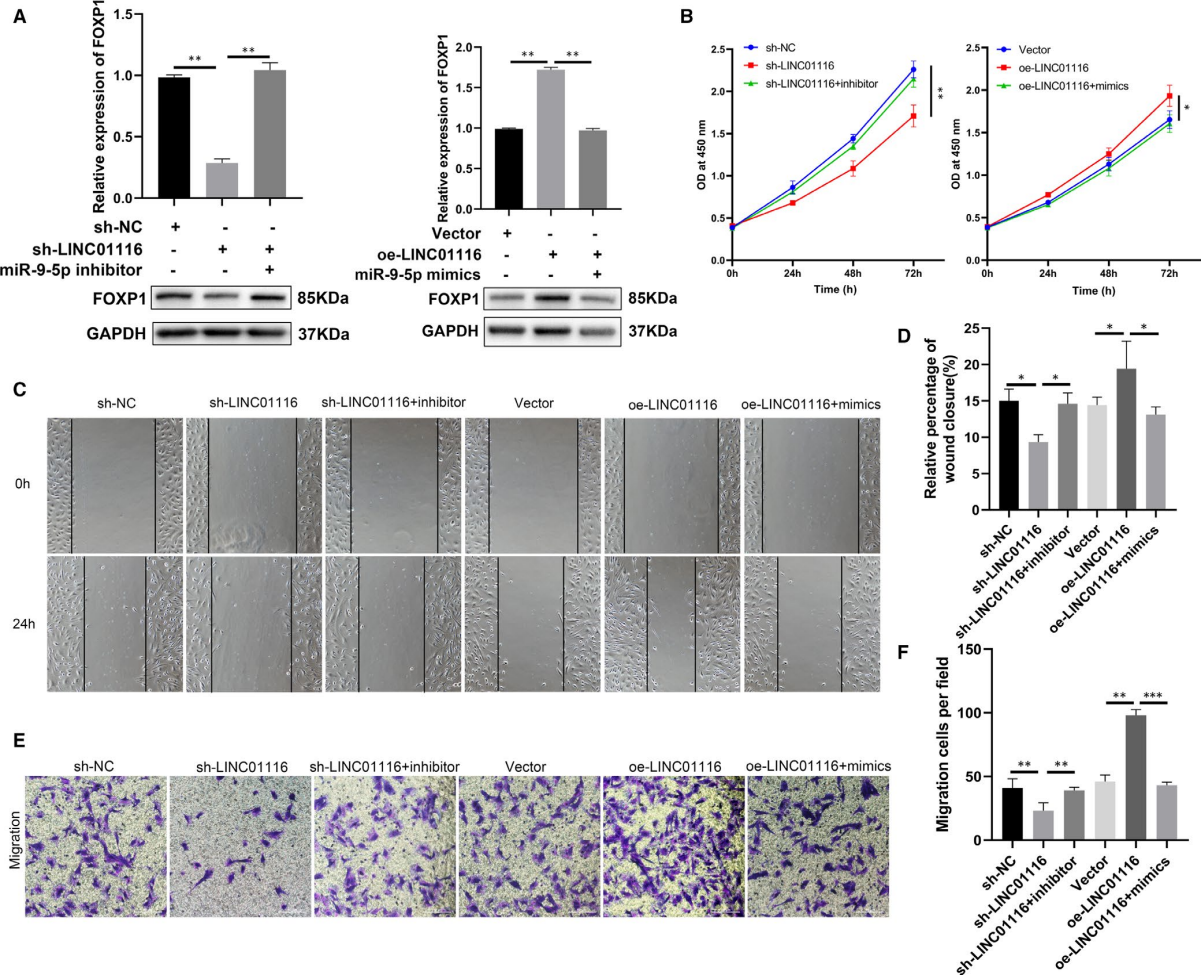
LINC01116 promotes ESCs proliferation and migration through LINC01116/miR‐9‐5p/FOXP1 axis. A, Relative mRNA level of FOXP1 by qRT‐PCR (up) and protein level of FOXP1 by Western blot (down) in ESCs transfected with indicated sh‐NC, sh‐LINC01116, inhibitor, vector, oe‐LINC01116, or mimics. (B) CCK‐8 assays carried out to assess the proliferation ability of ESCs transfected with indicated sh‐NC, sh‐LINC01116, inhibitor, vector, oe‐LINC01116, or mimics. (C to F) Cell migratory capabilities assessed by wound healing and trans‐well assays in ESCs transfected with indicated sh‐NC, sh‐LINC01116, inhibitor, vector, oe‐LINC01116, or mimics. Data are shown as mean ± SD, *P < .05, **P < .01, ***P < .001

To further demonstrate the role of LINC01116/miR‐9‐5p/FOXP1 axis in endometriosis, a series of rescue experiments were performed. The qRT‐PCR and Western blot results showed that knockdown of LINC01116 decreased the mRNA and protein levels of FOXP1 in ESCs, while overexpression of LINC01116 enhanced the levels of FOXP1 (Figure 6A). Moreover, miR‐9‐5p mimics or the inhibitor could reverse the effects induced by knockdown or overexpression of LINC01116, respectively (Figure 6A). We performed CCK‐8, wound healing and trans‐well assays to determine whether the biological function of LINC01116 could be reversed by miR‐9‐5p mimics or inhibitor. The results indicated that the miR‐9‐5p inhibitor could reverse the inhibiting effects of proliferation and migration caused by the knockdown of LINC01116 in ESCs, while miR‐9‐5p mimics could reverse these effects caused by overexpression of LINC01116 (Figure 6B‐F). These results confirm that LINC01116 can serve as a miR‐9‐5p sponge to regulate FOXP1 expression, thus promoting the progression of endometriosis.

## 4 DISCUSSION

There are many hypotheses about the pathogenesis of endometriosis, such as the immune microenvironment, eutopic endometrial biological behaviour, inflammatory microenvironment, stem cell function and epigenetics , but none can adequately explain the onset of the disease.^6^ Non‐coding RNA (ncRNA) refers to nucleic acid sequences that do not encode proteins, including long non‐coding RNA (lncRNA), circular RNA (circRNA) and microRNA (miRNA).^38^ With the rapid development of high‐throughput RNA‐Seq technology, the regulatory role of ncRNA in cancers, cardiovascular diseases, neurodegenerative diseases, and many others has gradually become a research hotspot.^39^, ^40^, ^41^ In the same way, some studies have revealed the role of ncRNA in endometriosis. For instance, miR‐205‐5p was found to be a novel suppressor of endometriosis through activating the ERK/AKT pathway by sponging ANGPT2 in ESCs.^42^ Another study revealed that the up‐regulated lncRNA SNHG4 promoted the proliferation of endometrial stromal cells via regulation of c‐Met and was mediated by miR‐148a‐3p in endometriosis.^18^ LncRNA‐TC0101441, which was identified as a novel metastasis‐related lncRNA in cancer, was found to promote the migration of endometriotic cells in an extracellular vesicle‐mediated manner.^43^ These findings inspired us to study the underlying functions and mechanisms of lncRNAs in endometriosis.

In the present study, we investigated the different lncRNA expression profiles in endometriosis using high‐throughput RNA sequencing. A total of 142 differentially expressed lncRNAs were identified, including 50 up‐regulated and 92 down‐regulated lncRNAs. After verification by qRT‐PCR, LINC01116 was selected for further investigation as it was one of the most significantly up‐regulated lncRNAs in the ecEM of the endometriosis patients. We conducted a number of functional assays in ESCs to investigate the influence of LINC01116 dysregulation in endometriosis. The results revealed that knockdown of LINC01116 could suppress the proliferation and migration of ESCs whereas overexpression of LINC01116 had the opposite effects. Therefore, our findings demonstrate that LINC01116 promotes the progression of endometriosis.

LncRNAs may act as competing endogenous RNAs (ceRNAs) of miRNAs thus regulating the expression of target mRNAs at the post‐transcriptional level.^11^ Through bioinformatics analyses and luciferase reporter assays, miR‐9‐5p was identified to be a target miRNA of LINC01116. Previous studies have reported miRNAs involved in the establishment and progression of endometriosis. For example, our preliminary investigations demonstrated that miR‐96‐5p was able to interact with the TGF‐β/SMAD signalling pathway in endometrial cells via direct targeting of TGFBR1, whereas miR‐34c‐5p could suppress the progression of EMT through targeting the Notch signalling pathway in endometriosis.^44^, ^45^ In this study, we demonstrated that the expression of miR‐9‐5p was up‐regulated after knockdown of LINC01116 in ESCs and that the expression level of miR‐9‐5p was significantly down‐regulated in ecEM relative to euEM tissues.

In humans, miR‐9‐5p is transcribed from three genes: MIR9‐1 (chromosomes 1), MIR9‐2 (chromosomes 5), and MIR9‐3 (chromosomes 15), and has been reported to play a role in a cohort of diseases, especially cancer.^46^ MiR‐9‐5p is highly expressed in glioma and breast cancer cells and promotes cell migration and proliferation.^47^, ^48^ Whereas in ovarian and gastric cancer cells, miR‐9‐5p is lowly expressed and inhibits cell migration and proliferation.^49^, ^50^ Thus, the role of miR‐9‐5p seems to be cell‐type dependent and requires further investigation. In this study, we observed the inhibitory effects of miR‐9‐5p in endometriosis cells, and the inhibition of cell proliferation and migration caused by LINC01116 knockdown could partially be attenuated by miR‐9‐5p inhibitor.

It is well known that miRNAs bind with the 3′‐UTR of target mRNAs to regulate gene expression through either translational repression or mRNA degradation. FOXP1 was identified to be the target gene of miR‐9‐5p by bioinformatic analysis and dual‐luciferase reporter assays. FOXP1 belongs to the subfamily P of the forkhead box (FOX) transcription factor family, which is involved in a wide range of cancers.^51^ A previous study has shown that FOXP1 is up‐regulated in endometriosis and promotes cell proliferation, migration and fibrosis via the Wnt/β‐catenin signalling pathway.^37^ In line with this previous study, we found that FOXP1 was negatively regulated by miR‐9‐5p in ESCs. Functional experiments further demonstrated that LINC01116 sponged miR‐9‐5p to counteract the inhibitory effect on its target gene, FOXP1. Finally, rescue assays demonstrated that LINC01116 promoted ESCs proliferation and migration by regulating miR‐9‐5p/FOXP1 interaction in endometriosis.

As far as we know, this is the first study that thoroughly examines the expression, function and regulation of LINC01116 in endometriosis. Furthermore, this is also the first study that investigates the relationship between LINC01116 and miR‐9‐5p. Importantly, our study obtained higher purity primary ESCs using previously described methods which could be used as a better in vitro cell model for endometriosis. This makes our results more credible. There are some limitations to this study. Firstly, the sample size used for RNA‐seq in our study was relatively small and we only included rASRM stage III/IV cases. More patients with different stages of endometriosis need to be included to further confirm the clinical value of LINC01116. Secondly, the ecEM tissue samples were derived from cyst walls of ovarian endometriosis patients and it was difficult to distinguish endometrial tissue from ovarian tissue. Thirdly, further detection of LINC01116 expression in body fluids is also needed to make LINC01116 a possible biomarker for endometriosis. In the future, we need to further evaluate the possibility of using LINC01116 as a therapy target for endometriosis.

## CONFLICT OF INTEREST

The authors declare that they have no conflict of interest.

## AUTHOR CONTRIBUTION

**Liangyi Cui:** Conceptualization (lead); Data curation (lead); Formal analysis (lead); Investigation (lead); Methodology (lead); Project administration (lead); Supervision (equal); Validation (equal); Visualization (equal); Writing‐original draft (equal); Writing‐review & editing (equal). **Silei Chen:** Conceptualization (equal); Data curation (equal); Resources (equal); Software (equal). **Dandan Wang:** Conceptualization (equal); Data curation (equal). **Qing Yang:** Conceptualization (equal); Funding acquisition (lead); Project administration (equal); Writing‐review & editing (equal).

## Data Availability

All data generated or analysed during this study are included in this article.
