# Corrigendum

**DOI:** 10.1111/jcmm.17316

**Published:** 2022-05-07

In Cui et al.,^1^ there is one error in the 24 h wound‐healing assay results of ‘oe‐LINC01116+mimics’ group of Figure 6C. The correct figure is shown below. The authors confirm that all results and conclusions of this article remain unchanged.

**FIGURE 6:**
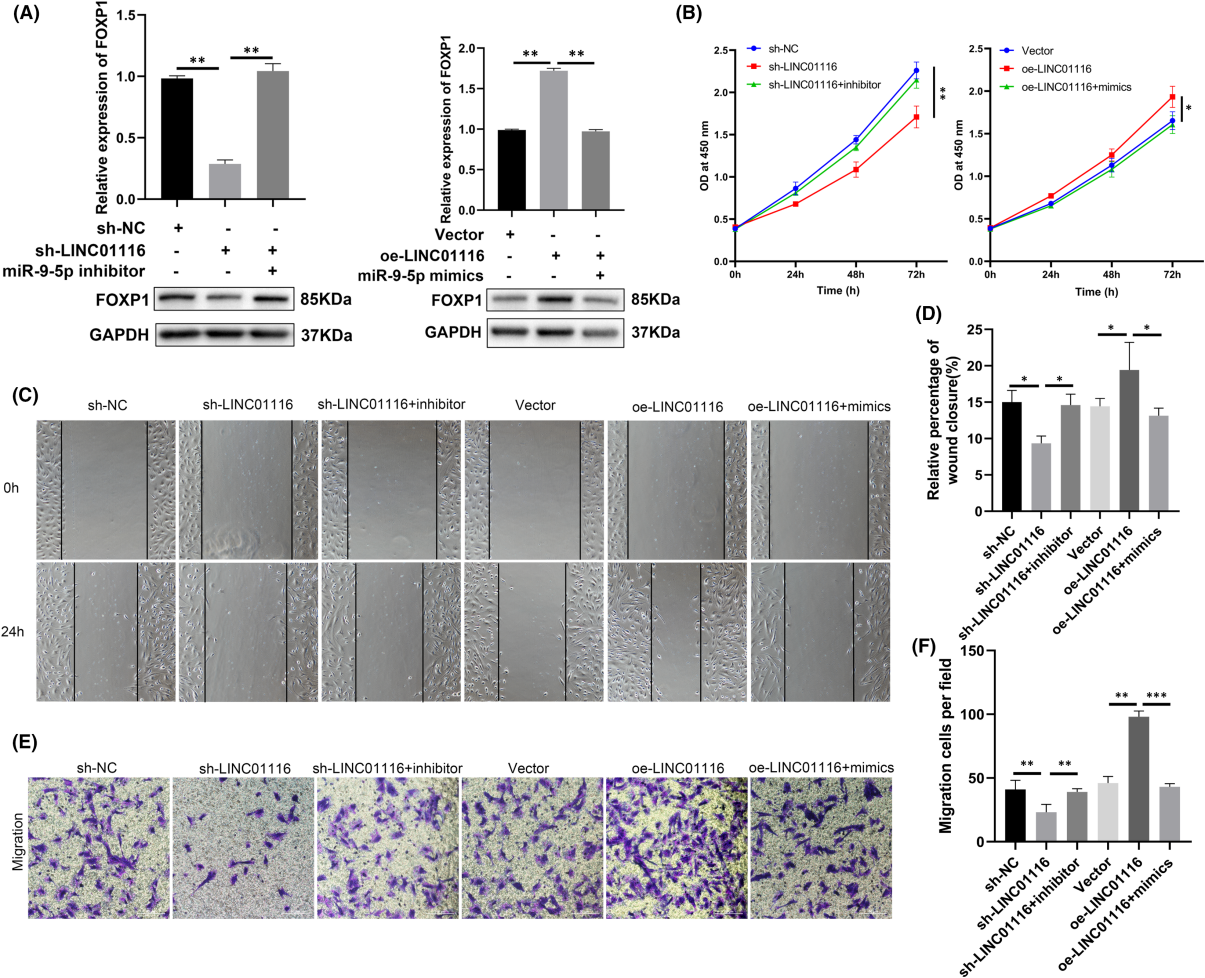
LINC01116 promotes ESCs proliferation and migration through LINC01116/miR‐9‐5p/FOXP1 axis. (A) Relative mRNA level of FOXP1 by qRT‐PCR (up) and protein level of FOXP1 by western blot (down) in ESCs transfected with indicated sh‐NC, sh‐LINC01116, inhibitor, vector, oe‐LINC01116, or mimics. (B) CCK‐8 assays carried out to assess the proliferation ability of ESCs transfected with indicated sh‐NC, sh‐LINC01116, inhibitor, vector, oe‐LINC01116, or mimics. (C–F) Cell migratory capabilities assessed by wound healing and trans‐well assays in ESCs transfected with indicated sh‐NC, sh‐LINC01116, inhibitor, vector, oe‐LINC01116, or mimics. The data are shown as mean ± SD, *p < 0.05, **p < 0.01, ***p < 0.00
